# Chronic corticosterone exposure causes anxiety- and depression-related behaviors with altered gut microbial and brain metabolomic profiles in adult male C57BL/6J mice

**DOI:** 10.1186/s13041-024-01146-x

**Published:** 2024-11-07

**Authors:** Hirotaka Shoji, Yasuhiro Maeda, Tsuyoshi Miyakawa

**Affiliations:** 1 Division of Systems Medical Science, Center for Medical Science, Fujita Health University, Toyoake, Aichi 470-1192 Japan; 2 Open Facility Center, Fujita Health University, Toyoake, Aichi 470-1192 Japan

**Keywords:** Corticosterone, Anxiety, Depression, Behavior, Gut microbiome, Metabolome, Mice

## Abstract

Chronic exposure to glucocorticoids in response to long-term stress is thought to be a risk factor for major depression. Depression is associated with disturbances in the gut microbiota composition and peripheral and central energy metabolism. However, the relationship between chronic glucocorticoid exposure, the gut microbiota, and brain metabolism remains largely unknown. In this study, we first investigated the effects of chronic corticosterone exposure on various domains of behavior in adult male C57BL/6J mice treated with the glucocorticoid corticosterone to evaluate them as an animal model of depression. We then examined the gut microbial composition and brain and plasma metabolome in corticosterone-treated mice. Chronic corticosterone treatment resulted in reduced locomotor activity, increased anxiety-like and depression-related behaviors, decreased rotarod latency, reduced acoustic startle response, decreased social behavior, working memory deficits, impaired contextual fear memory, and enhanced cued fear memory. Chronic corticosterone treatment also altered the composition of gut microbiota, which has been reported to be associated with depression, such as increased abundance of *Bifidobacterium*, *Turicibacter*, and *Corynebacterium* and decreased abundance of *Barnesiella*. Metabolomic data revealed that long-term exposure to corticosterone led to a decrease in brain neurotransmitter metabolites, such as serotonin, 5-hydroxyindoleacetic acid, acetylcholine, and gamma-aminobutyric acid, as well as changes in betaine and methionine metabolism, as indicated by decreased levels of adenosine, dimethylglycine, choline, and methionine in the brain. These results indicate that mice treated with corticosterone have good face and construct validity as an animal model for studying anxiety and depression with altered gut microbial composition and brain metabolism, offering new insights into the neurobiological basis of depression arising from gut-brain axis dysfunction caused by prolonged exposure to excessive glucocorticoids.

**Supplementary Information:**

The online version contains supplementary material available at 10.1186/s13041-024-01146-x.

## Introduction

Exposure to stress activates the hypothalamic–pituitary–adrenal (HPA) axis, triggering the secretion of steroid hormones or glucocorticoids (cortisol in humans and corticosterone in mice) from the adrenal cortex. Glucocorticoids bind to glucocorticoid receptors in the brain and are involved in physiological metabolism, immune responses, mood, and cognitive function [1–3]. Severe or prolonged stress can lead to HPA axis dysfunction and increased glucocorticoid levels, which have been implicated as significant risk factors in the pathogenesis of depression [4]. In mice, the administration of corticosterone (CORT) for a few weeks or more induces behavioral disturbances, which are reminiscent of the symptoms that characterize depression, such as increased anxiety-like and depression-related behaviors [5–17]. These findings suggest that mice chronically exposed to CORT are a valid animal model for studying depression.

The gut microbiota exerts a significant influence on various physiological processes in the host, such as the immune response, tryptophan metabolism, and neurotransmitter production [18–21], and is linked to behavior, affecting HPA responses and CORT levels [22–26]. Several lines of evidence have suggested that gut microbiome diversity is associated with neuropsychiatric and neurodegenerative disorders, including anxiety and depression [27]. Animal studies have shown that exposure to stress affects gut microbial composition [28, 29], and that chronic CORT treatment induces gut microbial changes, such as an increase in the abundance of the phylum Firmicutes and a decrease in the abundance of the phylum Bacteroidetes in cecal contents [30, 31]. Such CORT-induced changes in gut microbial composition may be a risk factor for behavioral disturbances and brain dysfunction, thus being considered a biomarker and a potential therapeutic target for depression. In animal facilities, various environmental factors can influence microbial composition [32]. Inter-facility variation could differentiate microbiome-disease associations, indicating the need for replicating findings from microbiome studies at multiple facilities [33].

Metabolomic profiling is a powerful tool for quantitatively measuring metabolic responses to normal or pathophysiological conditions and for uncovering significant biochemical signatures in diseases. A recent systematic review suggested that metabolomic changes in the blood and brain are implicated in the pathophysiology of depression [34]. Alterations in lipid metabolism in the liver were observed in mice chronically exposed to CORT, as indicated by elevated levels of oleic acid and palmitic acid [30]. Furthermore, a previous study demonstrated that rats administered chronic CORT showed changes in energy, lipid, and amino acid metabolism in whole brain samples [35]. The prefrontal cortex (PFC), hippocampus, habenula (Hb), paraventricular nucleus of the thalamus (PVT), and hypothalamus (Hypo), are among the brain regions associated with depression [36–39], However, what metabolomic changes in the specific brain regions are caused by chronic CORT exposure remain unknown.

In the present study, to evaluate mice chronically treated with CORT as an animal model of depression and to advance the understanding of the impact of chronic CORT exposure on brain function and mechanisms underlying depression, we investigated behavioral, gut microbial, and blood and brain metabolomic profiles of CORT-treated mice. There is a paucity of research on the effects of long-term exposure to CORT on some domains of behavior in mice, such as social behavior and learning and memory. Thus, we first investigated broad domains of behavior in adult male C57BL/6J mice chronically treated with CORT or vehicle (Veh) via drinking water for more than 4 weeks through 15 different behavioral tests to assess sensory and motor functions, locomotor activity in novel and familiar environments, anxiety-like and depression-related behaviors, social behaviors in novel and familiar environments, prepulse inhibition, working memory, spatial memory, and contextual and cued fear memory. Next, we measured plasma CORT levels to confirm the elevation of CORT after chronic oral administration of exogenous CORT. Additionally, we measured the weights of the adrenal glands, which play a critical role in CORT synthesis and secretion, as well as the thymus and spleen, which are involved in immune function, to assess the response of peripheral organs to repeated exogenous CORT administration. To examine the effects of CORT on the gut microbial composition, fecal samples were assessed using 16S rRNA gene amplicon sequencing. Finally, we investigated the plasma and brain metabolomic profiles of CORT- and Veh-treated mice. In addition to the HPA axis dysfunction hypothesis, there are various pathophysiological hypotheses for depression, such as monoamine deficiency, excitatory/inhibitory imbalance, and mitochondrial dysfunction hypotheses [40–43]. Therefore, in the metabolomic analysis, we sought to explore the concentrations of monoamines, glutamate and gamma-aminobutyric acid (GABA), amino acids, and some other metabolites in the brain regions associated with depression, including the PFC, Hb/PVT, hippocampus, hypothalamus, and plasma of chronic CORT- and Veh-treated mice.

## Materials and methods

### Animals

Male C57BL/6J mice 7 weeks old were purchased from the Jackson Laboratory Japan, Inc. (Kanagawa, Japan). After arrival at our animal facility, the mice were group-housed (four per cage) in plastic cages (250 × 182 × 139 mm; CLEA Japan, Inc., Tokyo, Japan) with paper chips for bedding (Paper Clean; Japan SLC, Inc., Shizuoka, Japan) covered with stainless-steel wire lids and filter caps (CLEA Japan, Inc., Tokyo, Japan). Rooms were maintained under a 12-h light/dark cycle (lights on at 7:00 h) at 23 ± 2 °C. All animals were provided with food (CRF-1; Oriental Yeast Co., Ltd., Tokyo, Japan) and water *ad libitum* throughout the experiments. More than 2 weeks after arrival, oral administration of corticosterone solution or vehicle was initiated. All experimental procedures were approved by the Institutional Animal Care and Use Committee of Fujita Health University.

### Corticosterone treatment

Corticosterone (95%, FUJIFILM Wako Pure Chemical Co., Japan) was dissolved in ethanol (99.5%, FUJIFILM Wako Pure Chemical Co., Japan) at 10 mg/mL and then mixed with filtered drinking water to a final concentration of 0.1 mg/mL CORT in 1% ethanol. Mice were divided into two groups: corticosterone (CORT)- and vehicle (Veh)-treated. The CORT-treated group had ad libitum access to a bottle of CORT solution. The Veh-treated group was given a water bottle containing a vehicle solution (1% ethanol in filtered drinking water). The solution in the bottle was changed every 3–7 days. Oral administration was continued until the end of the study, except for the sucrose preference test, as described below.

### Behavioral tests

Four weeks after the beginning of CORT administration, CORT-treated mice (n = 20) and Veh-treated mice (n = 20) were subjected to a battery of behavioral tests in the following order (see Additional file 1: Table S1 for the test order and age of animals): general health and neurological screen, light/dark transition, open field, elevated plus maze, hot plate, social interaction, rotarod, startle response/prepulse inhibition, Porsolt forced swim, three-chamber social approach, T-maze spontaneous alternation, tail suspension, contextual and cued fear conditioning, sucrose preference, and home cage social interaction tests, as previously described [44, 45]. In addition, another group of mice received CORT or Veh solution for more than 4 weeks and were used for the Barnes maze test to assess spatial memory (CORT, n = 5; Veh, n = 10). After each test, the floors and walls of the test apparatus were cleaned with a 70% ethanol solution and hypochlorous acid water to prevent bias based on olfactory cues. Except for the sucrose preference test, behavioral tests were performed between 9:00 and 17:00.

### General health and neurological screen

Physical characteristics, body weight, and rectal temperature were recorded. Neuromuscular strength was assessed by the grip strength and wire hang tests. Forelimb grip strength was measured using a grip strength meter (O’Hara & Co., Tokyo, Japan). The mice were lifted by their tails to grasp a wire grid with their forelimbs. They were then gently pulled back until they released the grid. The peak force of grip strength was recorded in Newtons (N). In the wire hang test, the mice were placed on a wire mesh (O’Hara & Co., Tokyo, Japan), which was inverted gently so that the subject grasped the wire. The latency to fall from the wire was recorded with a 60 s cut-off time.

### Light/dark transition test

The light/dark transition test, developed by Crawley and colleagues [46] to assess anxiety-like behavior, was performed as previously described [47]. The apparatus consisted of a cage (21 × 42 × 25 cm) divided into two equal chambers by a partition with a door (O’Hara & Co., Tokyo, Japan). One chamber had white plastic walls and was brightly lit (390 lx) by lights mounted above the ceiling of the chamber. The other chamber had black plastic walls and was dark (2 lx). Both chambers had a white plastic floor. Mice were placed in the dark chamber and allowed to move freely between the two chambers for 10 min with the door open. Behavior was recorded using video cameras mounted on the ceiling. The distance traveled (cm), time spent in the light chamber (s), number of transitions, and latency to first enter the light chamber (s) were automatically calculated using the ImageLD program (see “Image analysis for behavioral test”).

### Open field test

The open field test was conducted in the open field apparatus with the VersaMax activity monitoring system (Accuscan Instruments, Columbus, OH, USA) to assess locomotor activity. The open field arena was made of acrylic with transparent walls and a white floor (40 × 40 × 30 cm). The floor of the center area, defined as 20 × 20 cm, was illuminated at 100 lx. Each mouse was placed in a corner of an open field and allowed to explore freely for 120 min. The distance traveled (cm), vertical activity (rearing measured by counting the number of photobeam interruptions), time spent in the center area (s), and stereotypic counts (beam-break counts for stereotyped behaviors) were calculated for each 5-min block.

### Elevated plus maze test

The elevated plus maze test to assess anxiety-like behavior [48] was conducted as previously described [49, 50]. The apparatus consisted of two open arms (25 × 5 cm) and two closed arms of the same size with 15-cm-high transparent walls and a central square (5 × 5 cm) connecting the arms (O’Hara & Co., Tokyo, Japan). The floor of the apparatus was made of white plastic plates and was elevated to 55 cm above the floor. The open arms were surrounded by a raised ledge (3-mm thick and 3-mm high) to prevent the mice from falling off the arms. Arms of the same type were placed opposite to one another. The illumination level in the central area was 100 lx. Each mouse was placed in the central square of the maze facing one of the closed arms. Distance traveled (cm), number of arm entries, percentage of entries into open arms, and percentage of time spent in open arms were measured using a video camera mounted above the apparatus during a 10-min test period. Data acquisition and analysis were performed automatically using the ImageEP program.

### Hot plate test

The hot plate test was used to assess pain sensitivity to a thermal stimulus. Each mouse was placed on a hot plate (55.0 ± 0.1 °C; Columbus Instruments, Columbus, OH, USA) and the latency to a paw response (s) was recorded with a cut-off time of 15 s. The paw response was defined as either a paw lick or a foot shake.

### Social interaction test

The social interaction test was performed to assess social behavior in a novel environment. Weight-matched mice (mean ± SD for differences in body weight between the two mice: CORT, 0.44 ± 0.22 g; Veh, 0.48 ± 0.23 g) from the same treatment group that had been housed in different cages were placed together in a white plastic box (40 × 40 × 30 cm; 100 lx at the center of the floor) together and allowed to explore it freely for 10 min. The total number of contacts, total duration of contacts (s), total duration of active contacts (measured when two mice made contact and one or both mice moved with a velocity of at least 10 cm/s), mean duration per contact (s), and total distance traveled (cm) were measured automatically using a video camera mounted above the apparatus and the image analysis program ImageSI.

### Rotarod test

Motor coordination and balance were assessed in the rotarod test. The mice were placed on a rotating drum (3 cm diameter, Accelerating Rotarod; Ugo Basile, Varese, Italy). They were subjected to three trials per day for 2 consecutive days. The latency to fall from the rod (s) was measured. The rotarod speed was accelerated from 4 to 40 rpm over 300 s.

### Three-chamber social approach test

The apparatus consisted of a rectangular, three-chambered box and a lid with a video camera (O’Hara & Co., Tokyo, Japan). Each chamber was made of white plastic (20 × 40 × 47 cm) and the partitions were made of transparent acrylic with a small square opening (5 × 3 cm). The three-chamber test was conducted as previously described [51]. In the first session, each test mouse was placed in the central chamber of the apparatus, which contained empty wire cages (9 cm in diameter and 11 cm in height, with vertical bars 0.5 cm apart) in the corners of each side chamber, and allowed to explore for 10 min (habituation session). Next, an unfamiliar C57BL/6J male mouse (stranger 1; 8–9 weeks old) that had no prior contact with the test mice was placed in the wire cage located in one of the side chambers. The location of the stranger mouse in the left and right chambers was systematically alternated between trials. First, the test mouse was placed in the central chamber and allowed to explore for a 10-min session to assess sociability (sociability test). Next, a second stranger mouse (stranger 2; 8–9 weeks old) was placed in the wire cage that had been empty during the first 10-min session to assess social preference for the new stranger (social novelty preference test). The test mouse had a choice between the first, already-investigated, now-familiar mouse (stranger 1) and the new, unfamiliar mouse (stranger 2). Time spent in each chamber and around each cage was automatically calculated from video images using the ImageCSI program.

### Acoustic startle response/prepulse inhibition test

Startle response and prepulse inhibition tests were conducted using a startle reflex measurement system (O’Hara & Co., Tokyo, Japan), as previously described [52]. Briefly, the mice were placed in a clear plastic cylinder and left undisturbed in a sound-attenuating chamber for 10 min. A loud sound stimulus (110 or 120 dB, white noise, 40 ms) was then presented as a startle stimulus. A prepulse sound stimulus (74 or 78 dB, white noise, 20 ms) was presented 100 ms before the startle stimulus to assess prepulse inhibition. A test session consisted of six trial types (i.e., two types of startle stimulus-only trials, and four types of prepulse inhibition trials: 74–110, 78–110, 74–120, and 78–120 dB). Six blocks of the six trial types were presented in a pseudorandom order, such that each trial type was presented once within a block for 10 min. The average interval was 15 s (range: 10–20 s). A 70-dB white noise was presented as background noise during the test. The peak amplitude of the startle response to the stimuli was recorded for 400 ms from the onset of the prepulse stimulus. The percentage PPI was calculated for each mouse using the following formula: percent PPI = 100 × [1 − (startle response amplitude in prepulse + startle trial)/(startle response amplitude in startle stimulus-only trial)].

### Porsolt forced swim test

The Porsolt forced swim test [53] was used to assess depression-related behavior. Mice were placed in a clear plastic cylinder (20 cm height × 10 cm diameter, O’Hara & Co., Tokyo, Japan) filled with water (approximately 21 °C) to a depth of 8 cm for a 10 min per day for 2 consecutive days. The percentage of immobility time was automatically recorded using the ImagePS/TS program as previously described [54, 55].

### T-maze spontaneous alternation test

The T-maze spontaneous alternation test was conducted to assess spatial working memory using a modified automatic T-maze apparatus (O’Hara & Co.), as previously described [44, 56]. The apparatus consisted of white plastic runways with 25-cm-high walls. It was partitioned into six areas: the stem of the T, a straight runway, the left and right arms, and the connecting passageways from the arms to the stem of the T. Mice were subjected to a session consisting of 10 trials per day for 3 days (a cut-off time of 50 min). Each trial consisted of a forced-choice run followed by a free-choice run (inter-trial interval, 60 s). In the forced-choice run, mice were forced to enter either the left or the right arm of the T-maze and were held in that arm for 10 s. After the 10 s, the doors of the passageway connecting the arm to the stem of the T were opened, and the mouse could return to the start compartment. A free-choice run began 3 s after the mice entered the start compartment. The mice were allowed to choose one of the arms in the free-choice run, and if the mice entered the arm opposite to the arm that the mice entered in the forced-choice run, the response was recorded as a correct response. Data acquisition and analysis were performed automatically using the ImageTM program (see “Image analysis for behavioral test”).

### Tail suspension test

The tail suspension test was performed to assess depression-related behavior [57]. Mice were suspended 30 cm above the floor in a visually isolated area, using adhesive tape placed approximately 1 cm from the tip of the tail. Immobility time was recorded for 10 min using the ImagePS/TS program in the same manner as for the forced swim test.

### Contextual and cued fear conditioning test

The contextual and cued fear conditioning test was conducted to assess fear memory using an automated video analysis system, as previously described [58]. First, mice were placed in a conditioning chamber (26 × 34 × 29 cm) and allowed to explore freely for 2 min. The animals were then presented with an auditory cue (55-dB white noise), which served as a conditioned stimulus (CS) for 30 s. During the last 2 s of the CS, the mice received a mild footshock (0.3 mA, 2 s) as an unconditioned stimulus (US). Two more CS–US pairings were presented at 120-s intervals. One day and 28 days after the conditioning session, a context test was performed in the conditioning chamber, in which the mice were allowed to explore for 5 min. A cued test in an altered context was then performed in a triangular box (35 × 35 × 40 cm) made of opaque white plastic located in another sound-attenuated room more than 3 h after the context test. In the cued test, after an initial 3-min period without CS presentation, the CS was presented during the last 3-min period. For each test, video images were recorded at one frame per second. Freezing time (%) and distance traveled (cm) were automatically measured in each trial using the ImageFZ program. Images were also recorded at a rate of 4 frames per second for 6 s from 2 s before the application of a 2-s footshock to 2 s after the footshock, and distance traveled (cm) was measured as an index of footshock sensitivity.

### Sucrose preference test

Mice were individually housed in plastic cages (250 × 182 × 139 mm) with fresh paper chips for bedding and were given two bottles of filtered tap water 7 days after the first contextual and cued fear conditioning test. The following day, the mice were given one bottle of water and a second bottle of 1% sucrose solution. The bottles were weighed at approximately 24-h intervals to measure water and sucrose intake over 4 days, with the left–right position changed daily. Sucrose preference was expressed as 100 × [(sucrose intake averaged over 4 days)/(sucrose intake averaged over 4 days + water intake averaged over 4 days)]. The mice were not given CORT or vehicle solutions in the test.

### Home cage social interaction test

The home cage social interaction test was conducted to assess social behavior and activity levels under familiar conditions in a home cage for 7 days. The social interaction monitoring system consisted of a home cage with paper chips as bedding and a cage top with an infrared video camera (25 × 15 × 23.5 cm, interior dimensions). Weight-matched mice of the same treatment group housed in separate cages, were housed together in the cage (mean ± SD for the differences in the body weight between the two mice: CORT, 0.50 ± 0.25 g; Veh, 0.42 ± 0.41 g). Video images at a rate of 1 frame per second were analyzed to assess social interaction by automatically counting the number of particles (animals) detected in each frame using the ImageHA program (one particle indicates contact between two mice, and two particles indicate that mice are not in contact with each other). Activity was quantified by measuring the number of pixels that changed between each pair of consecutive images. The mean number of animals and total activity level in each 1-h bin were calculated for 1 week.

### Barnes maze test

The Barnes circular maze test [59] was conducted to test spatial reference memory on “dry land,” a white circular surface, 1.0 m in diameter, with 12 holes equally spaced around the perimeter (O’Hara & Co., Tokyo, Japan). The circular open field was elevated 75 cm above the floor. A black Plexiglas escape box (17 × 13 × 7 cm) was located under one of the holes (target hole). In the acquisition session, two trials per day were conducted for 9 days. Each mouse was placed in the center of the maze in each trial and then allowed to explore it. If the mouse did not enter the escape box for a maximum of 5 min, it was gently guided to the escape hole. After entering the escape hole, the mouse remained in the escape box for 30 s before returning to the holding cage. The target’s location was consistent for a given mouse but randomized across mice. The maze was rotated daily, with the target’s spatial location unchanged for distal visual cues, to prevent bias based on olfactory or proximal cues. The latency to first reaching the target hole (s), number of errors to reach the target hole, distance traveled first to reach the target hole (cm), and number of omissions (visit to the target hole without subsequent entry into the target hole) were recorded automatically by the ImageBM program. One day and 28 days after the last acquisition session, probe trials were performed without the escape box to assess spatial reference memory. In the probe test, the time spent around each hole (s) was measured using the ImageBM program.

### Image analysis for behavioral test

Image analysis programs (ImageLD/EP/SI/CSI/PS/TS/TM/BM/FZ/HA) were used to analyze mouse behaviors automatically, as previously described [47, 49, 56, 58]. The programs, based on the public domain ImageJ software (developed by Wayne Rasband at the National Institute of Mental Health, Bethesda, US), were modified by Tsuyoshi Miyakawa. The ImageLD/EP/TM/FZ programs can be freely downloaded from the “Mouse Phenotype Database” (http://www.mouse-phenotype.org/).

### Assessment of food and solution intake

Food intake was measured weekly by weighing the food pellets in each cage (CORT, n = 5 cages; Veh, n = 5 cages) for 8 weeks from the beginning of the CORT and Veh treatments. Mean food intake (g) per mouse per day was calculated. Water bottles were also weighed in each cage, and mean solution intake (g) per mouse per day was calculated.

### Measurement of adrenal gland, thymus, and spleen weights

Adrenal glands, thymus, and spleen were collected from an independent cohort of mice after 6 weeks of treatment with CORT or vehicle (CORT, n = 10; Veh, n = 12). Organs were weighed immediately after collection. Relative weight of each organ was calculated by dividing the organ weight (mg) by body weight (g).

### Plasma corticosterone measurement

Mice treated with CORT or vehicle for 4 weeks (CORT, n = 12; Veh, n = 16) were used to assess stress response by measuring plasma CORT levels after the tail suspension test. Seven CORT-treated mice and eight Veh-treated mice were subjected to the tail suspension test for 10 min. Immediately after the test, A total of 200 µL of blood was collected from the facial or submandibular veins within 30 s of holding the mouse using a Goldenrod Animal Lancet (MEDIpoint, Inc., NY, USA). Similarly, blood was collected from the remaining mice that were experimentally naïve immediately after they were taken from their home cages. In our previous study, basal CORT levels measured from blood samples using the Animal Lancet [50, 60] were similar to those measured from trunk blood collected immediately after cervical dislocation and decapitation under stress-free conditions [61]. Thus, it is unlikely that the use of Animal Lancet influences CORT levels. Blood samples were placed into tubes containing 10 µL of heparin/saline solution (100 Units/mL) and temporarily stored on ice. They were then centrifuged at 3000×*g* for 10 min at 4 °C. Supernatants were collected and stored at − 80 °C until measurement. Plasma CORT concentrations were determined according to the manufacturer’s protocol, using an enzyme immunoassay kit (Assay Designs Inc., MI, USA).

### Fecal microbiome analysis

Fecal samples were collected from the colon of an independent group of mice (CORT, n = 8; Veh, n = 8) after cervical dislocation after 5 weeks of treatment. The samples were placed in tubes and stored at − 80 °C. DNA from fecal samples was extracted by a bead-based method, as previously described [62]. Prokaryote universal primers (Pro341F and Pro805R) with the sample-specific 8-bp dual-index barcode sequences were used to amplify V3 and V4 regions of 16S rDNA genes by polymerase chain reaction [62, 63]. The barcoded amplicons were paired-end sequenced on the Illumina MiSeq platform using the MiSeq Reagent Kit v3 (600 cycles, 2 × 284-bp cycle; Illumina, San Diego, CA, USA). Paired-end reads were joined using the fastq-join program [64]. The joined reads with a quality value score ≥ 20 for > 99% of the sequence were extracted using FASTX-Toolkit [65] and were used for further analysis. The chimeric sequences were removed using uSearch61 software [66, 67]. Taxonomy assignment from the sequence reads was performed using Metagenome@KIN software (World Fusion, Tokyo, Japan) and the database RDP MultiClassifier ver.2.11 [68] with an 80% confidence level. The amplicon sequencing and taxonomic assignment were performed by TechnoSuruga Laboratory Co., Ltd. (Shizuoka, Japan).

The read counts were analyzed for assessing α-diversity (species richness and evenness from the rarefied counts; observed number of microbial genera, Chao1 index, and Shannon indexe) using R packages ‘vegan’ (ver. 2.5-7) [69]. In addition, β-diversity was visualized with principal coordinate analysis (PCoA) with Bray–Curtis dissimilarity and the percentage of variance explained by the PCoA axis was calculated using the pcoa function in the R software package ‘ape.’ Permutational multivariate analysis of variance (PERMANOVA) was used to assess the differences using the adonis function in the R package ‘vegan.’ Furthermore, the relative abundance of the microbiota at the genus level was analyzed using the linear discriminant analysis (LDA) effect size (LEfSe) method [70] to identify taxonomic features characterizing the differences between the two treatment groups (LDA score > 3, p < 0.05).

### Plasma and brain metabolomic analysis

Eight CORT-treated and seven Veh-treated mice were euthanized by cervical dislocation on the 40th day of treatment. Immediately after decapitation, approximately 300 µL of trunk blood was collected in a tube containing heparin solution and temporarily stored on ice until plasma collection. Brains were removed and placed on an ice-cold glass plate to minimize possible metabolic changes. The medial prefrontal cortex (mPFC), habenula, and paraventricular nucleus of the thalamus (Hb/PVT), dentate gyrus (DG) and CA of the hippocampus, and hypothalamus (Hypo) were quickly dissected, collected in tubes, frozen in liquid nitrogen, and then stored at − 80 °C in a freezer until use (for the weight of brain tissue, see Table S2). The procedure from picking up the mouse to freezing the brain samples took approximately 10 min for each mouse.

Concentrations of metabolites were determined by high-performance liquid chromatography–tandem mass spectrometry (HPLC–MS/MS). All steps of tissue sample extraction were performed on ice. To brain samples was added 10 μmol/L 2-morpholinoethanesulfonic acid (internal standard) in methanol (500 μL for 50 mg of tissue). The samples were homogenized using an ultrasonic homogenizer (THU-80; AS ONE Co., Osaka, Japan), and water (250 μL for 50 mg of tissue) and chloroform (400 μL for 50 mg of tissue) were then added. After the mixture was centrifuged at 4 °C and 15,000×*g* for 15 min, the supernatant was filtered using a 10 kDa cutoff filter (Amicon Ultra centrifugal filter; Merck Millipore, Bulington, MA, USA). The filtrate was lyophilized and the precipitate was dissolved in 50 μL of water, after which it was subjected to HPLC–MS/MS (LCMS-8060; Shimadzu Co., Kyoto, Japan). Metabolites were eluted from a reverse phase column (Discovery HS F5, 150 × 2.1 mm, 3 μm; Supelco, Bellefonte, PA, USA) with the gradient method using a mobile phase with 0.1% formic acid in water and 0.1% formic acid in acetonitrile at a flow rate of 0.25 mL/min, and were then analyzed in electronspray ionization (ESI) positive or negative ion multiple reaction monitoring (MRM) mode of MS/MS. The Q1 or Q3 pre bias voltages, collision energies and mass transitions used are listed in Table S3. The MS–MS data of metabolites were analyzed using principal component analysis (PCA) and partial least square-discriminant analysis (PLS-DA) to visualize group differences in the metabolites. Metabolites that contribute to group classification were selected using the criteria of a variable importance in projection (VIP) score of the PLS-DA greater than 1.0, and an unadjusted p-value of less than 0.05, which have been used as a widely accepted standard in metabolomic studies. The selected metabolites were used for enrichment (over-representation) analysis through MetaboAnalyst 5.0 [71].

### Statistical analysis

Statistical analyses were performed using SAS Studio (SAS OnDemand for Academics; SAS Institute, Cary, NC, USA) and R (version 3.6.3). Behavioral data were analyzed using Student’s t-test or two-way repeated-measures ANOVAs with treatment as a between-subject variable and trial/time/block as a within-subject variable. When a treatment × trial/time/block interaction was significant, simple main effect analyses were conducted to examine the treatment effect at each time point. Microbial and metabolite abundances were analyzed by the Mann–Whitney U test and Student’s t-test using SAS and R packages described above. Comparisons of organ weights and plasma corticosterone levels between treatment groups were performed using Student’s t-test or two-way ANOVA. Statistical significance level was set at 0.05. Values in graphs are expressed as mean ± SEM.

## Results

### Physical characteristics and neurological functions in CORT-treated mice

The battery of behavioral tests was performed after 4 to 14 weeks of CORT treatment (see Additional file 1: Table S1). The statistical results of behavioral data from CORT- and Veh-treated mice are summarized in Table S4.

**Fig. 1:**
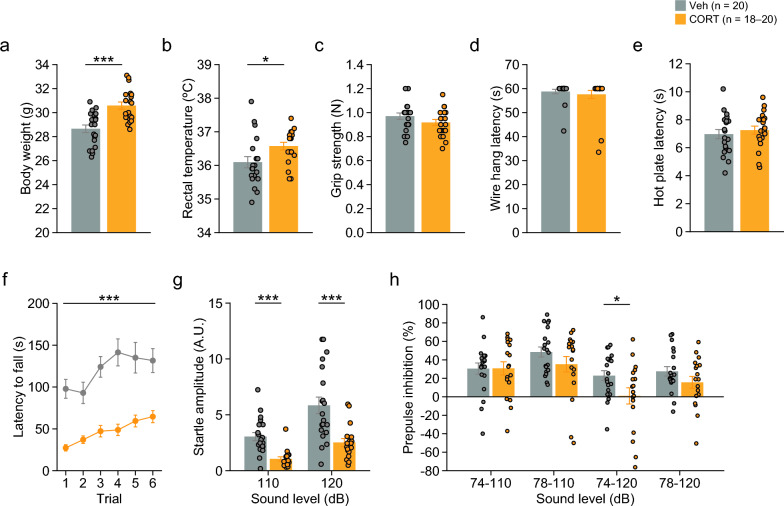
Body weight, body temperature, muscular strength, sensitivity to thermal stimulus, motor function, and prepulse inhibition in mice chronically treated with corticosterone. **a** Body weight (g), **b** body temperature (°C), **c** grip strength (Newton, N), **d** latency to fall off the wire (s) in the wire hang test, **e** latency to response to thermal stimulus (s) in the hot plate test, **f** latency to fall off the rod in the rotarod test, **g** acoustic startle response to loud noise (110 and 120 dB white noise), and **h** prepulse inhibition of the startle response with a prepulse of either 74 or 78 dB white noise in CORT- and Veh-treated mice. Values are means ± SEM. *p < 0.05. ***p < 0.001

CORT-treated mice showed increased body weight (Fig. 1a: 4-week treatment, t_38_ = 4.44, p < 0.0001; Additional file 2: Fig. S1a: 12-week treatment, t_32_ = 3.65, p = 0.0009), increased food intake (Additional file 2: Fig. S1b; F_1,8_ = 35.77, p = 0.0003; interaction, F_7,56_ = 4.12, p = 0.0010), and solution intake (Additional file 2: Fig. S1c; F_1,8_ = 131.96, p < 0.0001; interaction, F_7,56_ = 10.56, p < 0.0001) compared to Veh-treated mice. CORT-treated mice showed increased rectal temperature after 4 weeks of treatment (Fig. 1b: t_38_ = 2.39, p = 0.0218). Reduced rotarod latency was observed in CORT-treated mice (Fig. 1f: treatment, F_1,38_ = 34.66, p < 0.0001; interaction, F_5,190_ = 1.36, p = 0.2411), suggesting that chronic CORT treatment caused motor dysfunction or decreased motivation to perform the task. There were no significant effects of treatment on grip strength (Fig. 1c: t_38_ = 1.44, p = 0.1594), wire hang latency (Fig. 1d: t_38_ = 0.62, p = 0.5394), or hot plate latency (Fig. 1e: t_38_ = 0.66, p = 0.5160), indicating no differences in muscular strength and thermal pain sensitivity between the treatment groups. The prepulse inhibition test showed that acoustic startle responses at 110 and 120 dB sound stimuli and prepulse inhibition of the startle response at 74–120 dB trials were lower in CORT-treated mice than in Veh-treated mice (Fig. 1g, h: 110 dB, t_36_ = 4.74, p < 0.0001; 120 dB, t_36_ = 3.89, p = 0.0004; 74–110 dB, t_36_ = 0.03, p = 0.9770; 78–110 dB, t_36_ = 1.35, p = 0.1855; 74–120 dB, t_36_ = 2.18, p = 0.0361; 78–120 dB, t_36_ = 1.46, p = 0.1534).

### Increased anxiety-like and depression-related behaviors in CORT-treated mice

**Fig. 2:**
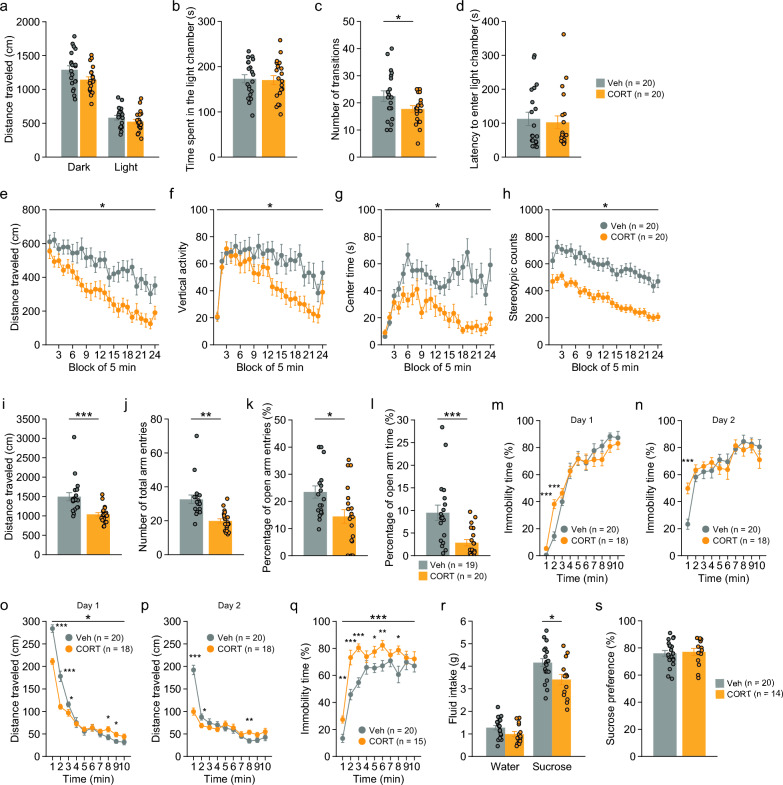
Locomotor activity and anxiety-like and depression-related behaviors in mice chronically treated with corticosterone. **a**–**d** Light/dark transition test: **a** distance traveled (cm) in the dark and light chambers, **b** time spent in the light chamber (s), **c** number of transitions, and **d** latency to enter the light chamber (s). **e**–**h** Open field test: **e** distance traveled (cm), **f** vertical activity, **g** time spent in the center area (s), and **h** stereotypic counts. **i**–**l** Elevated plus maze test: **i** total distance traveled (cm), **j** number of total arm entries, **k** percentage of open arm entries (%), and **l** percentage of time spent in the open arms. **m**–**p** Porsolt forced swim test: **m**, **n** percentage of immobility time (%) and **o**, **p** distance traveled (cm) in ten 1-min blocks of the test session on test days 1 and 2. **q** Percentage of immobility time (%) in the tail suspension test. **r**, **s** Sucrose preference test: **r** water and sucrose solution intake (g) and **s** percentage of sucrose preference (%). Values are means ± SEM. *p < 0.05. **p < 0.01. ***p < 0.001

CORT-treated mice showed a lower number of transitions between the light and dark chambers than Veh-treated mice in the light/dark transition test (Fig. 2c: t_38_ = 2.03, p = 0.0497), while there were no statistically significant differences in the distance traveled in the dark and light chambers (Fig. 2a: dark, t_38_ = 2.01, p = 0.0515; light, t_38_ = 1.21, p = 0.2354), time spent in the light chamber (Fig. 2b: t_38_ = 0.21, p = 0.8319), and latency to enter the light chamber (Fig. 2d: t_38_ = 0.37, p = 0.7147) between treatment groups. In the open field test, CORT-treated mice exhibited significant reductions in distance traveled (Fig. 2e: F_1,38_ = 11.20, p = 0.0019), vertical activity (Fig. 2f: F_1,38_ = 5.19, p = 0.0284), time spent in the center area (Fig. 2g: F_1,38_ = 25.27, p < 0.0001), and stereotypic counts (Fig. 2h: F_1,38_ = 35.53, p < 0.0001) compared to Veh-treated mice. In the elevated plus maze test, CORT-treated mice also showed decreases in distance traveled (Fig. 2i: t_37_ = 4.05, p = 0.0003), number of total arm entries (Fig. 2j: t_37_ = 4.56, p < 0.0001), percentage of open arm entries (Fig. 2k: t_37_ = 2.67, p = 0.0112), and percentage of time on open arms (Fig. 2l: t_37_ = 3.70, p = 0.0007) compared to Veh-treated mice. These results suggest that chronic CORT treatment decreases locomotor activity and increases anxiety-like behavior.

In the forced swim test, there were significant treatment × time interactions for the percentage of immobility time (Fig. 2m: treatment, F_1,36_ = 0.05, p = 0.8186; interaction, F_9,324_ = 3.09, p = 0.0014) and distance traveled (Fig. 2o: treatment, F_1,36_ = 4.82, p = 0.0346; interaction, F_9,324_ = 13.06, p < 0.0001) on day 1. Similar results were found on day 2 for immobility (Fig. 2n: treatment, F_1,36_ = 0.39, p = 0.5372; interaction, F_9,324_ = 2.49, p = 0.0093) and distance traveled (Fig. 2p: treatment, F_1,36_ = 2.90, p = 0.0973; interaction, F_9,324_ = 12.27, p < 0.0001). Then, simple main effect analysis revealed that CORT-treated mice exhibited increased immobility compared to Veh-treated mice in the first and second time bin on day 1 (all p < 0.001) and in the first time bin on day 2 (p < 0.0001), and CORT-treated mice also showed a significantly shorter distance traveled than Veh-treated mice on days 1 and 2 (Fig. 2o, p: p < 0.05). In addition, increased immobility in CORT-treated mice was observed in the tail suspension test (treatment, F_1,33_ = 24.08, p < 0.0001; interaction, F_9,297_ = 2.08, p = 0.0309) in several time bins during the 10-min test (Fig. 2q: p < 0.05). In the sucrose preference test, CORT-treated mice consumed a decreased volume of sucrose solution compared to Veh-treated mice (Fig. 2r: t_32_ = 2.63, p = 0.0132), suggesting anhedonia-like behavior, although there were no statistically significant differences in water intake (Fig. 2r: t_32_ = 1.92, p = 0.0644) and the percentage of sucrose preference (Fig. 2s: t_32_ = 0.34, p = 0.7361) between treatment groups. Together, these data indicate increased depression-related behavior in CORT-treated mice.

### Decreased social behavior in CORT-treated mice

**Fig. 3:**
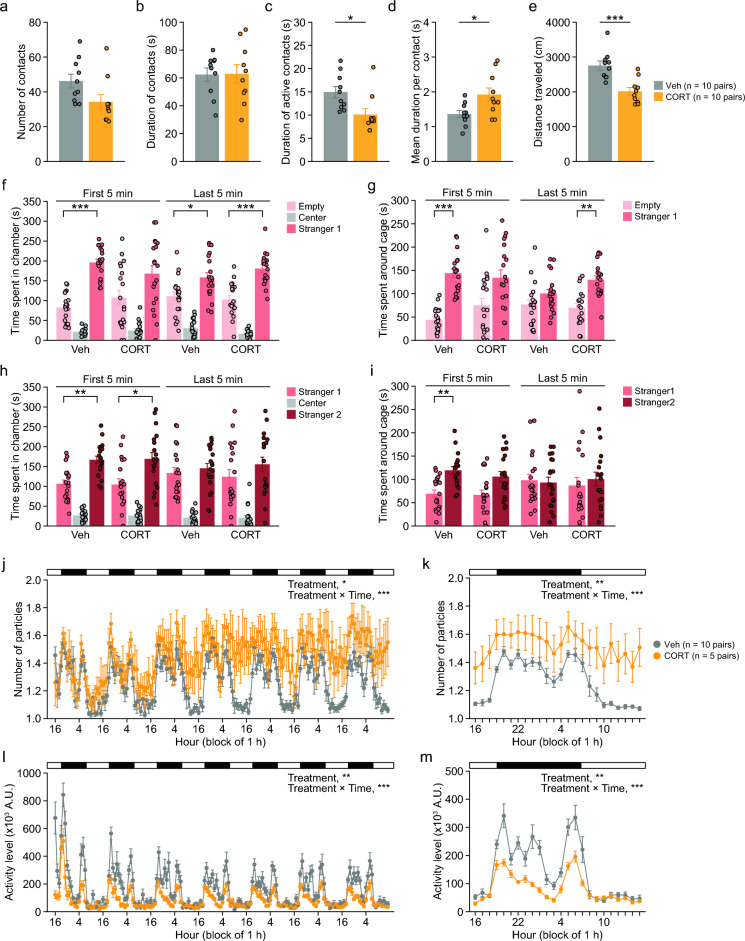
Social behavior of mice chronically treated with corticosterone. **a**–**e** Social interaction test: **a** number of contacts, **b** duration of contacts (s), **c** duration of active contacts (s), **d** mean duration per contact (s), and **e** total distance traveled (cm). **f**, **g** Three-chamber sociability test: **f** time spent in chambers (s) and **g** time spent around the cage (s). **h**, **i** Three-chamber social novelty preference test: **h** time spent in the chamber (s) and **i** time spent around the cage (s). **j**–**m** Home cage social interaction test: **j** number of particles (two particles indicated that the mice were not in contact with each other, and one particle indicated contact between the two mice) in 1-h bin for 7 days, **k** number of particles averaged over the last 3 days, **l** activity level in 1-h bin for 7 days, and **m** activity level averaged over the last 3 days. Values are means ± SEM. *p < 0.05. **p < 0.01. ***p < 0.001

In the social interaction test, two mice from different cages in the same treatment group were placed in a novel chamber. CORT-treated mice exhibited a trend toward a decreased number of contacts (Fig. 3a: t_18_ = 2.10, p = 0.0504), a shorter duration of active contacts (Fig. 3c: t_18_ = 2.70, p = 0.0146), and a shorter distance traveled (Fig. 3e: t_18_ = 4.32, p = 0.0004) compared to Veh-treated mice. These data indicate that chronic CORT treatment decreased the frequency of social interaction, although the contact duration per contact was increased (Fig. 3d: t_18_ = 2.58, p = 0.0189). There was no significant treatment effect on the total duration of contacts (Fig. 3b: t_18_ = 0.06, p = 0.9542).

In the three-chamber sociability test, Veh-treated mice spent a longer time in the chamber with the cage containing stranger 1 than in the chamber containing an empty cage (Fig. 3f: t_19_ = 6.87, p < 0.0001), and spent a longer time around the cage containing stranger 1 than around the empty cage (Fig. 3g: t_19_ = 7.23, p < 0.0001), whereas CORT-treated mice showed no significant differences between the time spent in the chambers (Fig. 3f: t_18_ = 1.58, p = 0.1314) and time around cages (Fig. 3g: t_18_ = 1.93, p = 0.0692) during the first 5-min period. These results indicate that CORT-treated mice displayed no preference for a novel mouse. During the last 5-min period of the sociability test, the preference for chamber or cage containing stranger 1 was observed in both Veh-treated mice (for time in chamber, t_19_ = 2.13, p = 0.046; for time around cage, t_19_ = 1.38, p = 0.1814) and CORT-treated mice (for time in chamber, t_18_ = 4.12, p = 0.0006; for time around cage, t_18_ = 3.83, p = 0.0012).

In the three-chamber social novelty preference test, Veh-treated mice spent a longer time in the chamber and around the cage with stranger 2 than with stranger 1 during the first 5-min period (Fig. 3h, i: for time in chamber, t_19_ = 3.34, p = 0.0034; for time around cage, t_19_ = 3.38, p = 0.0031). Similar results were found in CORT-treated mice during the first 5-min period (Fig. 3h and i: for time in chamber, t_18_ = 2.13, p = 0.0464; for time around cage, t_18_ = 1.94, p = 0.0673). During the last 5-min period, there were no significant differences between the time spent in the chamber with stranger 2 and the time spent in the chamber with stranger 1, or between the time spent around the cage containing stranger 2 and the time spent around the cage containing stranger 1, in both Veh-treated mice (for time in chamber, t_19_ = 0.45, p = 0.6547; for time around cage, t_19_ = 0.21, p = 0.8294) and CORT-treated mice (chamber, t_18_ = 0.86, p = 0.3972; for time around cage, t_18_ = 0.48, p = 0.6365).

Pairs of mice from different cages of the same treatment group that had not been encountered previously were housed together in a cage and left for 7 days to measure locomotor activity and social interaction in a home cage. In the home cage social interaction test, there were significant main effects of treatment and significant treatment × time interactions in the number of particles (one particle indicates contact between the two mice, and two particles indicate that the mice are not in contact with each other) (Fig. 3j: treatment, F_1,13_ = 7.56, p = 0.0166; interaction, F_167,2171_ = 1.94, p < 0.0001) and the activity level (Fig. 3l: treatment, F_1,13_ = 15.63, p = 0.0017; interaction, F_167,2171_ = 3.15, p < 0.0001). To assess social interaction following an acclimation in the cage, the number of particles and activity levels were averaged over the last 3 days. There were significant main effects of treatment and significant treatment × time interactions in the average number of particles (Fig. 3k: treatment, F_1,13_ = 9.17, p = 0.0097; interaction, F_23,299_ = 2.51, p = 0.0002) and the average activity level (Fig. 3m: treatment, F_1,13_ = 11.94, p = 0.0043; interaction, F_23,29_9 = 4.37, p < 0.0001). A significantly greater number of particles was found in CORT-treated mice than in Veh-treated mice in the time period of 16–19, 21, 23, 2–4, and 8–15 (Fig. 3k: p < 0.05). Lower activity levels were observed in CORT-treated mice in the time period of 19, 20, 22–1, and 5–7 (Fig. 3m: p < 0.05). These data indicate that CORT-treated mice exhibited reduced social contacts and decreased activity in the familiar environment.

### Decreased spontaneous alternation behavior in CORT-treated mice

**Fig. 4:**
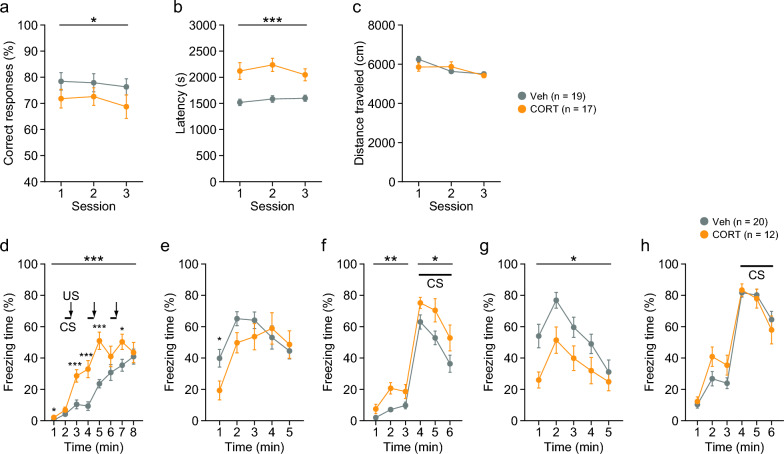
Spontaneous alternation and fear conditioning in mice chronically treated with corticosterone. **a**–**c** T-maze spontaneous alternation test: **a** correct responses (%), **b** latency to complete a session (s), and **c** total distance traveled (cm) to complete a session. **d**–**h** Fear conditioning test: freezing time (%) in the conditioning session on day 1 (**d**), context test on day 2 (**e**), cued test on day 2 (**f**), context test on day 29 (**g**), and cued test on day 29 (**h**). Values are means ± SEM. *p < 0.05. **p < 0.01. ***p < 0.001

CORT-treated mice displayed reduced spontaneous alternation, as indicated by the lower percentage of correct responses compared to Veh-treated mice in the T-maze test (Fig. 4a: F_1,34_ = 4.26, p = 0.0466), which is suggestive of reduced working memory. CORT-treated mice took a longer time to complete a session than Veh-treated mice (Fig. 4b: F_1,34_ = 18.29, p = 0.0001). There was no significant difference in the distance traveled to complete the session between the treatment groups (Fig. 4c: F_1,34_ = 0.23, p = 0.6370).

### Altered fear-related behavior in CORT-treated mice

During the conditioning session of the fear conditioning test, CORT-treated mice exhibited higher freezing (Fig. 4d: treatment, F_1,30_ = 13.64, p = 0.0009; interaction, F_7,210_ = 4.36, p = 0.0002) and shorter distance traveled (Additional file 3: Fig. S2a: treatment, F_1,30_ = 13.06, p = 0.0011; interaction, F_7,210_ = 1.82, p = 0.0845) than Veh-treated mice. The increased freezing was observed during the first minute and from the third to seventh minutes of the conditioning session before and after CS–US presentation, which indicates the possibility of suppressed exploration or increased anxiety in the novel environment and increased fear to footshock in CORT-treated mice. During the 2-s period of US presentation, CORT-treated mice traveled shorter distances than Veh-treated mice (Additional file 3: Fig. S2f–h: for US1, F_1,30_ = 5.42, p = 0.0268; for US2, F_1,30_ = 3.48, p = 0.0719; for US3, F_1,30_ = 8.70, p = 0.0061), suggesting decreased sensitivity to footshock in CORT-treated mice.

One day after the conditioning, CORT-treated mice showed significantly lower freezing (Fig. 4e: treatment, F_1,30_ = 1.00, p = 0.3251; interaction, F_4,120_ = 2.83, p = 0.0276) and greater distance traveled (Additional file 3: Fig. S2b: treatment, F_1,30_ = 5.75, p = 0.0229; interaction, F_4,120_ = 5.07, p = 0.0008) than Veh-treated mice in the context test. Similar results were observed 28 days after the conditioning for freezing (Fig. 4g: treatment, F_1,30_ = 7.35, p = 0.0110; interaction, F_4,120_ = 1.07, p = 0.3725) and distance traveled (Additional file 3: Fig. S2d: treatment, F_1,30_ = 9.05, p = 0.0053; interaction, F_4,120_ = 1.39, p = 0.2401) in the same context. These data raise two possibilities: an increase in escape response to the context and a reduction in both recent and remote contextual fear memory in CORT-treated mice.

In the cued test conducted 1 day after the conditioning, increased freezing was observed in CORT-treated mice during the pre-CS period (Fig. 4f: treatment, F_1,30_ = 12.63, p = 0.0013; interaction, F_2,60_ = 2.34, p = 0.1053) and during the CS period (Fig. 4f: treatment, F_1,30_ = 5.59, p = 0.0247; interaction, F_2,60_ = 0.24, p = 0.7865). In addition, CORT-treated mice traveled longer distance than Veh-treated mice during the pre-CS period (Additional file 3: Fig. S2c: treatment, F_1,30_ = 11.64, p = 0.0019; interaction, F_2,60_ = 3.11, p = 0.0518) and during the CS period (Additional file 3: Fig. S2c: treatment, F_1,30_ = 4.29, p = 0.0470; interaction, F_2,60_ = 0.11, p = 0.8966). There were no significant differences in freezing and distance traveled between the treatment groups during the pre-CS and CS periods in the cued test 28 days after the conditioning (Fig. 4h and Additional file 3: Fig. S2e). These observations suggest that chronic CORT treatment induced generalized fear and enhanced cued fear memory at least 1 day after the conditioning in CORT-treated mice, and that CORT-induced behavioral changes did not last for 4 weeks after the conditioning.

### No deficits in spatial reference memory in CORT-treated mice

**Fig. 5:**
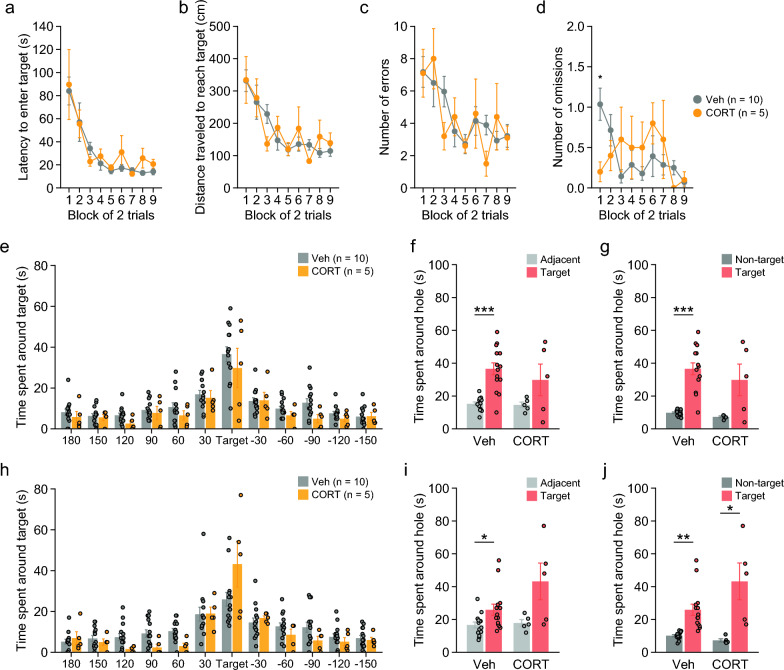
Spatial memory in mice chronically treated with corticosterone. **a**–**j** Barnes maze test: **a** latency to enter the target hole (s), **b** distance traveled to first reach the target hole (s), **c** number of errors to first reach the target hole, and **d** number of omissions across the acquisition session. **e**–**g** Probe trial performed 1 day after the last acquisition session: **e** time spent around each hole (s), **f** time spent around the target and adjacent holes (s), and **g** time spent around the target and non-target holes (s). **h**–**j** Probe trial tested 28 days after the last acquisition session: **h** time spent around each hole (s), (i) time spent around the target and adjacent holes (s), and **j** time spent around the target and non-target holes (s). Values are means ± SEM. *p < 0.05. **p < 0.01. ***p < 0.001

In the acquisition session of the Barnes maze test, there were no significant main effects of treatment and no significant treatment × time interactions on the latency to reach the target hole (Fig. 5a: treatment, F_1,17_ = 0.32, p = 0.5781; interaction, F_8,136_ = 0.31, p = 0.9608), distance traveled to reach the target hole (Fig. 5b: treatment, F_1,17_ = 0.03, p = 0.8582; interaction, F_8,136_ = 0.81, p = 0.5931), and number of errors to reach the target hole (Fig. 5c: treatment, F_1,17_ = 0.03, p = 0.8717; interaction, F_8,136_ = 0.88, p = 0.5356). In the first acquisition session, CORT-treated mice showed a significantly lower number of omissions than Veh-treated mice (Fig. 5d: treatment, F_1,17_ = 0.07, p = 0.7953; interaction, F_8,136_ = 2.18, p = 0.0331), suggesting that CORT-treated mice had increased motivation to escape from the maze.

In the probe tests conducted 1 day and 28 days after the acquisition session, there was no significant effect of treatment on time spent around the target hole between CORT- and Veh-treated mice (Fig. 5e, h: for probe test 1, t_17_ = 0.82, p = 0.4230; for probe test 2, t_17_ = 2.01, p = 0.0601). Veh-treated mice spent more time around the target hole than around the adjacent holes (Fig. 5f, i: for probe test 1, t_13_ = 5.85, p < 0.0001; for probe test 2, t_13_ = 2.42, p < 0.0307) and around the non-target holes (Fig. 5g, j: for probe test 1, t_13_ = 6.67, p < 0.0001; for probe test 2, t_13_ = 4.00, p = 0.0015). CORT-treated mice also spent a significantly longer time around the target hole than around the non-target holes in the probe test 2 (Fig. 5g, j: for probe test 1, t_4_ = 2.23, p = 0.0891; for probe test 2, t_4_ = 3.00, p = 0.0398), although there were no significant differences between the time spent around the target hole and the mean time spent around the adjacent holes (Fig. 5f, i: for probe test 1, t_4_ = 1.50, p = 0.2090; for probe test 2, t_4_ = 1.92, p = 0.1275). These results indicate that CORT-treated mice exhibited no obvious deficits in spatial reference memory.

### Increased plasma CORT levels in CORT-treated mice

One-half of the mice treated with CORT or vehicle for 4 weeks were subjected to the tail suspension test to measure stress-induced levels of plasma CORT. CORT-treated mice exhibited greater immobility than Veh-treated mice (Additional file 4: Fig. S3a: F_1,13_ = 12.39, p = 0.0038; interaction, F_9,117_ = 0.82, p = 0.6032). Immediately after the test, blood was collected from the tested mice and the remaining mice that were not subjected to the test. Two-way ANOVA showed a significant main effect of treatment and no significant interaction in CORT levels (Additional file 4: Fig. S3b: treatment, F_1,24_ = 5.33, p = 0.0299; test, F_1,24_ = 0.39, p = 0.5401; interaction, F_1,24_ = 2.60, p = 0.1197). Post-hoc analysis indicated that CORT-treated mice had higher plasma CORT levels than Veh-treated mice, although there was no significant difference in plasma CORT levels between the two treatment groups subjected to the tail suspension test (t_13_ = 0.62, p = 0.5469) or between Veh-treated mice subjected to the behavioral test and CORT-treated mice that were not tested (t_11_ = 0.97, p = 0.3510).

### Decreased weights of adrenal glands, thymus, and spleen in CORT-treated mice

**Fig. 6:**
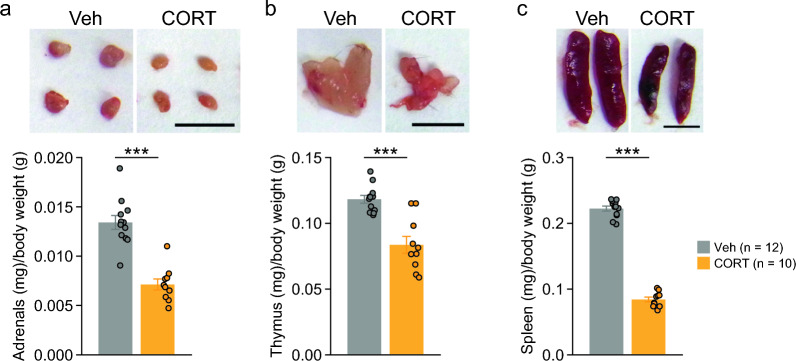
Organ weights of mice chronically treated with corticosterone. **a** Adrenal gland weight (mg)/body weight (g), **b** thymus weight (mg)/body weight (g), and **c** spleen weight (mg)/body weight (g) in CORT- and Veh-treated mice. Scale bars: 5 mm. Values are means ± SEM. ***p < 0.001

After 6 weeks of treatment, CORT-treated mice had significantly lower weights of the adrenal glands (Fig. 6a: t_20_ = 6.89, p < 0.0001), thymus (Fig. 6b: t_20_ = 5.22, p < 0.0001), and spleen (Fig. 6c: t_20_ = 26.31, p < 0.0001) than Veh-treated mice. These data indicate CORT-induced organ atrophy.

### Altered intestinal microbial composition in CORT-treated mice

**Fig. 7:**
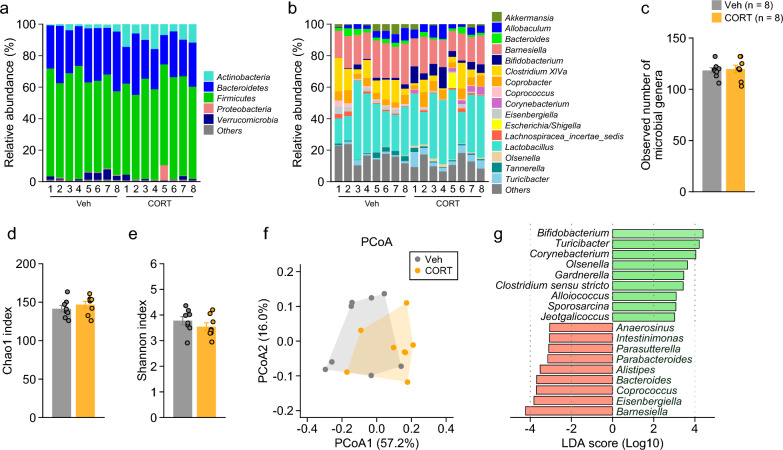
Intestinal microbial composition in mice chronically treated with corticosterone. **a**, **b** Relative abundance (%) of intestinal microbiota at the phylum and genus levels in each mouse (taxonomies with a relative abundance of less than 1% or not identified were included in ‘Others’). **c**–**e** Alpha diversity of the microbiota at the genus level: **c** observed number of microbial genera, **d** Chao1 index, and **e** Shannon index. **f** Beta diversity of the microbiota at the genus level was visualized using principal coordinate analysis (PCoA) with Bray–Curtis dissimilarity. **g** Taxa were differentially abundant between CORT- and Veh-treated mice (n = 8 in each group), which was identified by the linear discriminant analysis (LDA) effect size (LEfSe) method (LDA score > 3, p < 0.05). Values are means ± SEM

The abundant microbial taxa at the phylum level were *Firmicutes*, *Bacteroidetes*, and *Actinobacteria* (taxonomies with mean relative abundance above 1% in either group and ‘Others’ that are taxonomies with a relative abundance of less than 1% or not identified were shown in Fig. 7a; for details, see Additional file 1: Table S5). Statistical analysis revealed that CORT-treated mice showed a higher abundance of *Actinobacteria* and *Candidatus Saccharibacteria* than Veh-treated mice (Additional file 1: Table S5: χ^2^ = 10.59, p = 0.0011; χ^2^ = 7.08, p = 0.0078, respectively). At the genus level (Fig. 7b; for details, see Additional file 1: Table S6), there were no significant differences between the two treatment groups in the measures of alpha diversity: observed number of species (Fig. 7c: t_14_ = 0.30, p = 0.7668), Chao1 index (Fig. 7d: t_14_ = 0.87, p = 0.3979), and Shannon index (Fig. 7e: t_14_ = 1.07, p = 0.3044), which indicates that CORT- and Veh-treated mice have similar species richness and evenness in fecal samples. The beta diversity representing the dissimilarity of microbial composition between the two groups was visualized with principal coordinate analysis (PCoA) using Bray–Curtis dissimilarity (Fig. 7f), and permutational multivariate analysis of variance (PERMANOVA) showed significant group differences for Bray–Curtis dissimilarity (F_1,14_ = 4.19, p = 0.015). Further analysis using the linear discriminant analysis (LDA) effect size (LEfSe) method revealed that there were significant effects of treatment on 18 genera (LDA score > 3, p < 0.05), indicating differences in microbial composition that CORT-treated mice had increased abundances of 9 genera (*Bifidobacterium*, *Turicibacter*, *Corynebacterium*, *Olsenella*, *Gardnerella*, *Clostridium sensu stricto*, *Alloiococcus*, *Sporosarcina*, and *Jeotgalicoccus*) and decreased abundances of 9 genera (*Barnesiella*, *Eisenbergiella*, *Coprococcus*, *Bacteroides*, *Alistipes*, *Parabacteroides*, *Parasutterella*, *Intestinimonas*, and *Anaerosinus*) compared to Veh-treated mice (Fig. 7f).

### Chronic CORT treatment-induced metabolomic changes in the brain and plasma

**Fig. 8:**
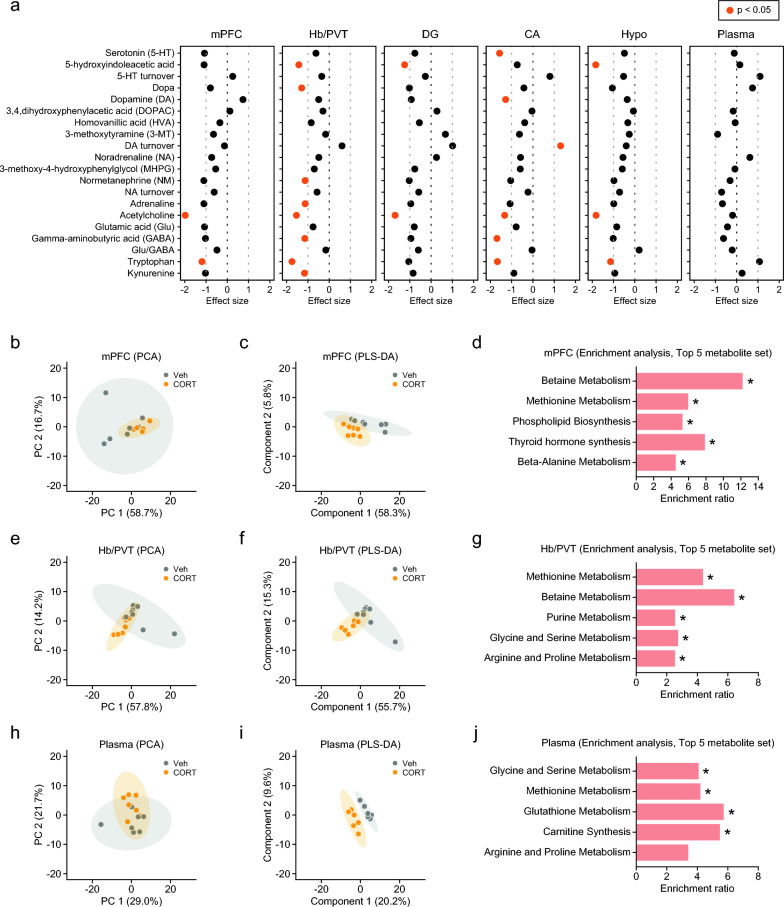
Brain and plasma metabolites in mice chronically treated with corticosterone. **a** Brain and plasma metabolites related to neurotransmission in CORT- and Veh-treated mice. **b**, **e**, **h** Principal component analysis (PCA) score plots of metabolomic data in CORT- and Veh-treated mice. **c**, **f**, **i** Partial least squares discriminant analysis (PLS-DA) score plots of metabolomic data. **d**, **g**, **j** Enrichment analysis of metabolites with a VIP value of > 1 in PLS-DA and p-value of < 0.05 in the t-test for comparisons between CORT- and Veh-treated mice. **b**–**d** Medial prefrontal cortex (mPFC). **e**–**g** Habenula/paraventricular nucleus of the thalamus (Hb/PVT). **h**–**j** Plasma. *p < 0.05

First, we investigated the effect of chronic CORT treatment on brain neurotransmitters, which is hypothesized to be associated with depression (Fig. 8a: for statistical results of monoamines, glutamate, GABA, other neurotransmitters, and tryptophan, which is a substrate for the synthesis of serotonin, see Additional file 1: Table S7). CORT-treated mice showed decreased levels of serotonin in the CA and its metabolite 5-hydroxyindoleacetic acid (5-HIAA) in the Hb/PVT, DG, and hypothalamus (Fig. 8a: all p < 0.05). CORT-treated mice also exhibited decreased serotonin and 5-HIAA levels in the mPFC, although the differences did not reach a significance level (Fig. 8a and Additional file 1: Table S7: p = 0.0585 and p = 0.0546, respectively). Lower levels of tryptophan were observed in the mPFC, Hb/PVT, CA, and hypothalamus of CORT-treated mice (Fig. 8a: all p < 0.05). In addition, CORT-treated mice showed decreased levels of dopamine in the CA, normetanephrine in the Hb/PVT, and acetylcholine in all the brain regions, and GABA in the Hb/PVT and CA, compared to Veh-treated mice (p < 0.05), while increased dopamine turnover was observed in the CORT-treated mice in the CA (Fig. 8a and Additional file 1: Tables S7 and S8: p < 0.05). In the plasma, there were no significant differences in neurotransmission-related molecules between CORT- and Veh-treated mice (Fig. 8a).

Next, metabolomic profiles of brain regions and plasma were visualized using principal component analysis (PCA) of metabolomic data from both CORT- and Veh-treated mice (Additional file 5: Fig. S4a). The PCA plot indicates differences in the metabolomic profiles between the brain and plasma. In each brain region and plasma, a visual inspection of the metabolites using PCA plots was conducted to investigate whether the CORT-treated group was separated from the Veh-treated group. The PCA plots of each brain region and plasma showed no clear separation between the treatment groups (Fig. 8b, e, h; Additional file 5: Fig. S4b, e, and h). PLS-DA revealed different metabolic patterns between the treatment groups (Fig. 8c, f, i, and Additional file 5: Fig. S4c, f, and i). In each brain region and plasma, lists of metabolites with VIP scores (VIP > 1.0) and p value of t-test for group comparison in each metabolite (p < 0.05) were used for enrichment (over-representation analysis, ORA) using MetaboAnalyst 5.0 (see Additional file 1: Table S8). The enrichment analysis showed that significantly enriched metabolite sets were betaine and methionine metabolism in each brain region, as indicated by decreased levels of adenosine, choline, cystathionine, glycine, dimethylglycine, methionine, and FAD in the brain regions of CORT-treated mice (Additional file 1: Table S8), and glycine and serine metabolism and methionine metabolism in plasma (Fig. 8c, f, i and Fig. S4d, g, j).

## Discussion

The present study showed that administration of CORT via drinking water for more than 4 weeks resulted in alterations across multiple aspects of behavior, including decreased locomotor activity, increased anxiety-like and depression-related behaviors, decreased social behavior in novel and familiar environments, reductions in acoustic startle response and prepulse inhibition, impaired working memory, reduced recent and remote contextual fear memory, and increased cued fear memory, but no significant changes in muscular strength and spatial reference learning and memory. Behavioral data demonstrate that mice chronically treated with CORT can be used as an animal model with high construct and face validity for studying anxiety and depression. Moreover, the current study revealed that long-term exposure to CORT leads to changes in intestinal microbial composition and metabolic shifts in both the plasma and brain. These findings imply a possible link between CORT-induced alterations in behavior and the gut–brain axis.

Basal plasma CORT levels in mice treated with CORT were comparable to those in vehicle-treated mice subjected to the tail suspension test. This suggests that the blood CORT levels induced by our oral administration regimen were equivalent to those triggered by stress in the test condition, which was designed to elicit depression-related behavior. The body weights of mice exposed to CORT by the administration regimen increased, which is consistent with the findings of previous studies [72–76]. Body weight gain is one of the physical features of depression, as observed in several mouse models of depression, such as the subchronic and mild social defeat stress model [77], chronic subordination stress model [78], postpartum depression model [55], and the SERT-deficient model [60]. Glucocorticoids induce lipolysis and proteolysis to provide energetic fuels for the stress response and contribute to fat storage and adipogenesis [79, 80], which might result in body weight gain. The increased body weight of CORT-treated mice may be partially due to hyperphagia, as indicated by their increased food and solution intake. In contrast, chronic exposure to CORT caused a decrease in the weight of the immune organs (thymus, spleen, and adrenal glands), as reported in previous studies [16, 73, 82, 83]. Considering that glucocorticoids have pleiotropic effects on the immune system [3, 84], atrophy of immune organs may reflect the possibility of a compensatory mechanism to the long-term elevation of CORT levels or dysfunction of the immune system that can render mice more vulnerable to anxiety- and depression-related behaviors.

Long-term exposure to CORT resulted in decreased locomotor activity and increased anxiety-like and depression-related behaviors, which is consistent with previous findings [5–17]. A number of reports have shown that mice chronically treated with CORT exhibit reduced sucrose preference or anhedonia-like behavior [10, 75, 85–87]. Similarly, in the present study, decreased sucrose intake was observed in CORT-treated mice. The results of these studies indicate that prolonged exposure to CORT leads to behavioral consequences that seem comparable to some of the core features of depression.

Chronic CORT treatment caused a slight decrease in social contacts, reduced duration of active contacts, and increased duration per contact in a novel environment of the social interaction test, which might be attributed to decreased locomotor activity. Some reports have showed a negative association between depressive symptoms and the amount of social interactions [88, 89], although others have reported no difference in the quantity of social interactions between participants with depressive symptomatology and controls [90–92]. With our knowledge, it remains unknown whether the increased mean duration per contact is observed in depressive patients. The behavioral characteristics, i.e., reduced duration of active contacts, increased mean duration per contact, and decreased distance traveled, were seen in 5-HTT−/− mice, which are considered as an animal model for depression [60]. These findings raise the possibility that the behavioral pattern may be commonly observed among mouse models for depression. CORT-treated mice also exhibited a decreased number of physical contacts in the familiar environment of the home cage social interaction test, suggesting reduced social interaction during the light and dark phases. The result of the home cage test may not be explained by basal locomotor activity, at least during the light phase, during which CORT- and Veh-treated mice did not differ in activity levels. Given the limited number of studies, more animal and human studies are needed to validate the association of depression and the behavioral traits in a dyadic social interaction. Reduced social behavior in CORT-treated mice has been reported in previous studies using the three-chamber sociability test and in the social interaction test adopted in the social defeat paradigm [75, 86, 93–95]. Consistent with previous reports in which social behavior was measured over a short period, that is, for 2.5 or 5 min, the present study indicates reduced social preference in CORT-treated mice during the first 5-min period of the sociability test. Our study further revealed no apparent impairment in social memory assessed using the social novelty preference test in the three-chamber paradigm. These findings suggest that long-term exposure to CORT disrupts the social approach in novel and familiar environments but causes no apparent deficits in social recognition memory.

Chronic administration of CORT induced cognitive deficits in a variety of behavioral paradigms for working memory in the Y-maze test [94–96], spatial memory in the Morris water maze test [97–100] and the Barnes maze test [97], and fear memory in the step-down and step-through passive avoidance test [95, 98, 101–105] and fear conditioning test [97, 106]. The Y-maze test has been conducted using a continuous trials procedure that allowed the animals to freely explore the three arms of the Y-maze [94–96]. The Y-maze continuous procedure has the possibility of inter-trial interference that may cause modest spontaneous alternation rates [107]. Additionally, if an animal has a side preference (e.g., always turning right) in the Y-maze, this will result in high alternation rates [107]. Thus, the continuous procedure can complicate the interpretation of behavioral consequences. The T-maze test used in the present study consisted of a forced-choice run followed by a free-choice run in each trial, with an inter-trial interval of 60 s, possibly reducing the influence of inter-trial interference. Our T-maze test results confirmed that chronic CORT treatment caused working memory deficits.

In contrast to a previous study [97], our study failed to find any significant behavioral differences between CORT- and Veh-treated mice in the Barnes maze test. The acquisition of spatial memory in the Barnes maze task with a large number of holes is considered difficult [108]. The maze apparatus utilized in a prior study by Darcet et al. [97] possessed a higher number of holes (20 in total) than that employed in the current study (12 holes), which might enable the former study to detect behavioral differences between the treatment groups. In the present study, a lower number of omissions to enter the escape box in the first acquisition session was observed in CORT-treated mice, suggesting that chronic CORT treatment increases the motivation to escape from the open space illuminated by bright lights. This aversive motivation might have contributed to the lack of deficits in the reference memory of CORT-treated mice, as demonstrated in this study.

CORT-treated mice exhibited decreased freezing in the context test and increased freezing in the cued test 1 day after conditioning, which is consistent with previous reports [97, 106]. The reduced freezing in the same context as that during conditioning may be explained by impaired reactivation and retrieval of contextual memory and/or increased escape responses to the conditioning context due to heightened fear in CORT-treated mice. Increased freezing during the pre-CS period of the cued test was context-independent, suggesting increased generalized fear in CORT-treated mice. Although the results of freezing in the context and cued tests appear to be somewhat contradictory, these findings may support the conclusion that long-term exposure to CORT is associated with an increase in fear responses. About 1 month after conditioning, CORT-treated mice displayed reduced freezing behavior during the context test, and there were no differences in freezing between CORT- and Veh-treated mice during the cued test. These results suggest impairments in long-term or remote fear memory in CORT-treated mice. Taken together, our data indicate that long-term exposure to CORT leads to various behavioral disturbances, including increased anxiety- and depression-like behaviors, reduced social behavior, and cognitive dysfunction, indicating that CORT-treated mice are a valid animal model for studying anxiety and depression.

Increasing evidence has shown that altered composition of the gut microbiota is associated with depression through the gut microbiota–brain axis [109–111]. In the present study, chronic CORT treatment induced changes in fecal microbial composition, as indicated by the increased abundance of the phyla *Actinobacteria* and *Candidatus Saccharibacteria* (formerly known as *TM7*) in the feces of CORT-treated mice, consistent with the findings of a recent study showing a similar trend for the two phyla in the cecal contents of CORT-treated mice [31]. Previous studies have reported that CORT-treated mice had an increased abundance of the phylum *Firmicutes* and a decreased abundance of the phylum *Bacteroidetes* in cecal contents [30, 31]. In our study, although CORT-treated mice exhibited a tendency toward a decrease in the abundance of *Bacteroidetes* compared to Veh-treated mice, no significant difference in the abundance of *Firmicutes* was observed between the treatment groups. These seemingly inconsistent results may be due to methodological differences in sampling sites (cecal contexts in previous studies and feces in the present study) and environmental conditions [112, 113] between the studies.

Our study further revealed differences in fecal microbiota at a more detailed taxonomic or genus level between CORT- and Veh-treated groups, as indicated by the increased abundance of *Bifidobacterium*, *Turicibacter*, *Corynebacterium*, and *Olsenella* and the decreased abundance of *Barnesiella*, *Eisenbergiella*, *Coprococcus*, *Bacteroides*, and *Alistipes* in CORT-treated mice. An increase or decrease in the abundance of *Bifidobacterium* has been observed in individuals with major depressive disorder [114, 115] and in stress-induced mouse models of depression [116, 117]. Some studies have indicated that the administration of *Bifidobacterium* species has beneficial and antidepressant effects [118, 119] and attenuates stress-induced increases in CORT levels [120, 121]; the increased abundance of *Bifidobacterium* may serve as a compensatory mechanism to counteract the behavioral and physiological effects of chronic CORT administration. The altered gut microbial composition caused by chronic exposure to CORT is partially similar to that observed in individuals with depression, as indicated by the increased abundance of *Olsenella* and decreased abundance of *Alistipes* and *Coprococcus* [115, 122–125]. In contrast to the microbial composition of CORT-treated mice, decreased abundance of *Corynebacterium* and increased abundance of *Eisenbergiella* were found in some animal models of chronic stress-induced depression [126–128], while decreased abundance of *Turicibacter* and increased abundance of *Barnesiella* and *Bacteroides* were observed in individuals with depression [115, 123, 125, 129, 130]. *Turicibacter* and *Corynebacterium* have been reported to increase or decrease in inflammatory bowel disease models [131–133] and diet-induced obesity models [134–137], suggesting that they can act as mediators of both pro-inflammatory and anti-inflammatory effects. Accumulating evidence indicates that increased activity in the immune system and inflammatory processes contribute to the development of depression [138–140] and that anti-inflammatory agents, including glucocorticoids, have antidepressant properties [139, 141]. These findings imply that chronic CORT-induced changes in the abundance of these genera, similar to *Bifidobacterium*, may be involved in the compensatory mechanism to inflammatory-related conditions, although the underlying mechanisms remain unknown. In this study, fecal microbiome was determined at the time point of 5 weeks following CORT exposure. Gut microbial composition can be affected by various environmental factors [32]. Thus, it is important to further explore the changes in gut microbial composition across different time points before and after CORT exposure under different environments to draw reliable conclusions regarding CORT-induced changes in gut microbiota. In addition, and further research is needed to clarify the precise role of the genera changed by elevated CORT conditions in behavioral and brain functions.

Our metabolic analysis revealed alterations in plasma glycine and serine metabolism, such as decreased glycine levels in CORT-treated mice. Glycine deficiency is associated with obesity and metabolic diseases [142]. Several studies have reported altered blood glycine levels in patients with depression, although the results have been inconsistent [143–146]. Glycine is degraded in the intestine by microbiota [147, 148]. Administration of *Lactobacillus paracasei* to the gut of germ-free mice results in decreased levels of glycine in the intestine [149]. These findings suggest that the peripheral metabolomic changes induced by CORT exposure could be mediated by alterations in gut microbial composition.

The present study showed decreased serotonin and 5-HIAA levels, accompanied by reduced tryptophan levels, in almost all brain regions in CORT-treated mice. CORT-treated mice also showed lower levels of dopamine, normetanephrine, and GABA in some brain regions. Decreased levels of neurotransmitters and their metabolites suggest chronic CORT-induced dysfunction in serotonergic, dopaminergic, noradrenergic, and GABAergic neurotransmission in the brain, which is consistent with the monoaminergic and GABAergic deficit hypothesis of depression [40, 150, 151]. Acetylcholine and monoamines in the brain contribute to attention, working memory, and other cognitive functions [152, 153]. Chronic CORT treatment decreased choline and acetylcholine levels in all the brain regions examined, which might be associated with impairments in PPI, fear memory, and working memory.

Enrichment analysis of the metabolomic data revealed alterations in betaine, methionine, and glycine metabolism induced by chronic CORT treatment in the brain, as indicated by decreased levels of choline, dimethylglycine, methionine, and glycine, which are commonly observed in the brain regions examined. Choline can be metabolized to betaine, which converts homocysteine to methionine, generating dimethylglycine metabolized to glycine, which is suggested to be an important nutrient for the prevention of chronic diseases and psychiatric disorders [154–157]. *S*-Adenosyl-methionine (SAMe), formed from methionine, has been used to treat depression [158]. In the present study, brain and plasma SAMe levels in CORT-treated mice were similar to those in Veh-treated mice. These results suggest that choline and methionine might be highly metabolized to maintain a normal level of SAMe in CORT-treated mice. Betaine improves the antidepressant effect of SAMe in patients with depression [159] and exerts an antidepressant effect in animal models of depression [160, 161]. Although this study did not measure betaine itself, the reduced metabolites of the betaine and methionine pathways suggest the possibility of betaine deficiency in CORT-treated mice. Consistent with a recent systematic review of metabolomic studies in animal models of depression, such as the chronic mild stress model, social defeat model, and LPS model [34], our findings suggest that decreased levels of metabolites for betaine and methionine metabolism as well as neurotransmission in the brain may be associated with a depressive state induced by chronic CORT exposure.

Accumulating evidence suggests that the gut microbiota can influence various nervous system functions via host metabolism, immune response, and neurotransmission [20, 25, 162]. Our study showed that chronic CORT treatment induced altered fecal microbial composition and plasma and brain metabolomic changes, which were accompanied by changes in a wide range of behaviors such as increased anxiety-like and depression-related behaviors, decreased social behavior, and disrupted working and fear memory. These findings suggest an association between gut microbial and metabolomic changes induced by CORT exposure and behavioral consequences relevant to depression. Some limitations should be taken into account in this study. First, ethanol solution was used as the vehicle. Ethanol consumption could affect basal levels of behavior, gut microbiota, and metabolites, which might mask or enhance the effects of corticosterone. Second, there is a lack of data on fecal metabolites. Fecal metabolome provides a functional readout of the gut microbiota [163], which could help us understand the relationship between CORT-induced changes in gut microbiota and plasma and brain metabolites. Third, our study did not include females. Sex differences in depression have been documented in humans, and its prevalence is twofold higher in women than in men [164, 165]. The use of both male and female animals is needed to comprehensively understand the effects of CORT exposure in the research of depression model. Finally, gut microbial analysis and metabolomics analysis were done using different cohorts of mice. Therefore, analysis of correlations between gut microbiota, metabolites, and behavior could not be performed to determine the relationship between the CORT-induced changes. Although further investigation is needed to reveal the causal link between chronic CORT-induced changes in the gut microbiota, metabolites, and behaviors, the findings of the present study provides important clues about the development of depression and potential targets for its treatment.

## Supplementary information

Additional file 1: Supplementary Table 1. A battery of behavioral tests in mice chronically treated with corticosterone. Supplementary Table 2. The weight of brain tissue used for metabolomic analysis. Supplementary Table 3. The Q1 or Q3 pre bias voltages, collision energies and mass transitions used in metabolomic analysis. Supplementary Table 4. Statistical analysis of behavioral data in mice chronically treated with corticosterone. Supplementary Table 5. Relative abundance of the intestinal microbiota at the phylum level in mice chronically treated with corticosterone. Supplementary Table 6. Relative abundance of the intestinal microbiota at the genus level in mice chronically treated with corticosterone. Supplementary Table 7. Neurotransmitters and their metabolites in the plasma and brain of mice chronically treated with corticosterone. Supplementary Table 8. Brain and plasma metabolites in mice chronically treated with corticosterone

Additional file 2: Supplementary Figure 1. Body weight and food and solution intake in mice chronically treated with corticosterone. **a** Body weight of mice treated with CORT for 12 weeks. **b** Mean food intake per mouse per day during the 8-week treatment period. **c** Mean solution intake per mouse per day during the 8-week treatment period. Values are means ± SEM. ***p < 0.001

Additional file 3: Supplementary Figure 2. Behavior in mice chronically treated with corticosterone. **a–e** Distance traveled in the fear conditioning test: **a** conditioning session on day 1, **b** context test on day 2, **c** cued test on day 2, **d** context test on day 29, and **e** cued test on day 29. **f–h** Distance traveled before, during, and after exposure to the first, second, and third US for 6 s in the conditioning session of the fear conditioning test. Values are means ± SEM. *p < 0.05

Additional file 4: Supplementary Figure 3. Plasma CORT levels in mice chronically treated with corticosterone. Approximately half of the CORT- and Veh-treated mice were subjected to the tail suspension test. The remaining half of the mice were left undisturbed in their home cages. **a** Percentage of immobility time in the TS test. **b** CORT levels in plasma taken from CORT- and Veh-treated mice that were either subjected to the TS or left undisturbed in HC. Values are means ± SEM. *p < 0.05. *p < 0.01

Additional file 5: Supplementary Figure 4. Brain metabolites in mice chronically treated with corticosterone. **a** Principal component analysis score plot of metabolomic data from the plasma and brain. **b, e, h** PCA score plots of metabolomic data from CORT- and Veh-treated mice. **c, f, i** Partial least squares discriminant analysis score plot of metabolomic data. **d, g, j** Enrichment analysis of metabolites with a VIP value of > 1 in PLS-DA and p-value of < 0.05 in the t-test for comparisons between CORT- and Veh-treated mice. **b–d** Dentate gyrus. **e–g** CA in the hippocampus. **h–j** Hypothalamus. *p < 0.05

## Data Availability

All the data used in this study are available from the authors upon request.
