# Supplementary figures and images for: Chronic corticosterone exposure causes anxiety- and depression-related behaviors with altered gut microbial and brain metabolomic profiles in adult male C57BL/6J mice
Source: Mol Brain. 2024 Nov 7;17:79. doi: 10.1186/s13041-024-01146-x (PMC11545877; doi:10.1186/s13041-024-01146-x)

**a** 12-week treatment

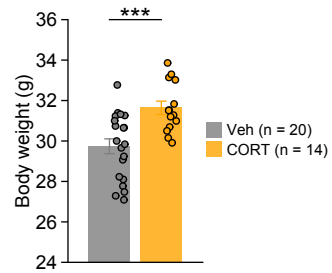

**b**

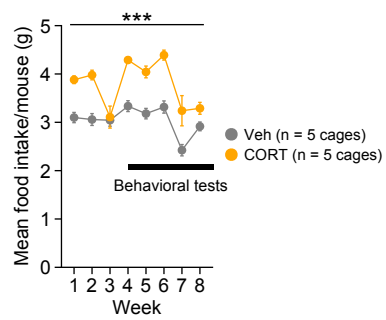

**c**

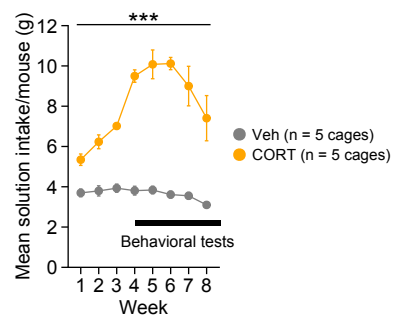

Supplement: Supplementary file 2 — Additional file 2: Supplementary Figure 1. Body weight and food and solution intake in mice chronically treated with corticosterone. a Body weight of mice treated with CORT for 12 weeks. b Mean food intake per mouse per day during the 8-week treatment period. c Mean solution intake per mouse per day during the 8-week treatment period. Values are means ± SEM. ***p < 0.001 [file 13041_2024_1146_MOESM2_ESM.pdf]

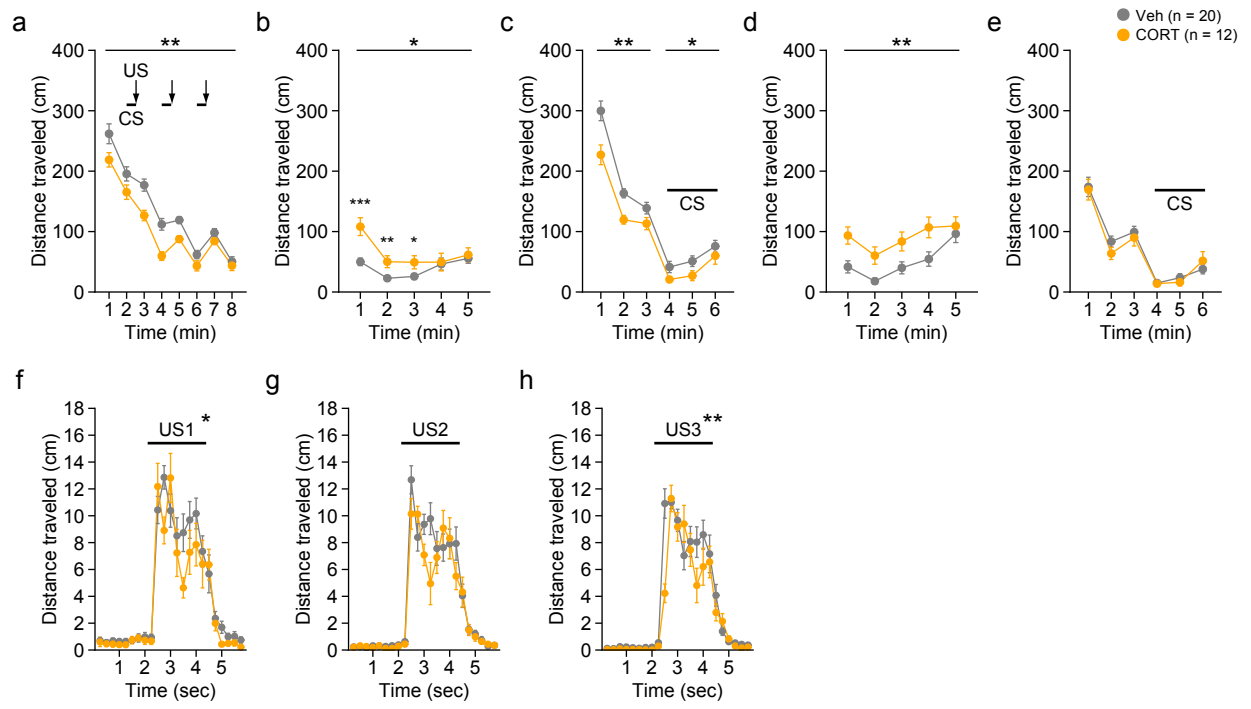

Supplement: Supplementary file 3 — Additional file 3: Supplementary Figure 2. Behavior in mice chronically treated with corticosterone. a–e Distance traveled in the fear conditioning test: a conditioning session on day 1, b context test on day 2, c cued test on day 2, d context test on day 29, and e cued test on day 29. f–h Distance traveled before, during, and after exposure to the first, second, and third US for 6 s in the conditioning session of the fear conditioning test. Values are means ± SEM. *p < 0.05 [file 13041_2024_1146_MOESM3_ESM.pdf]

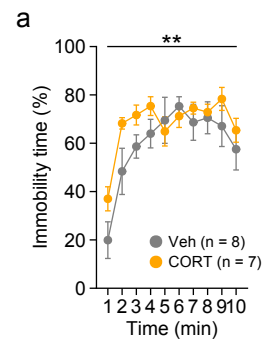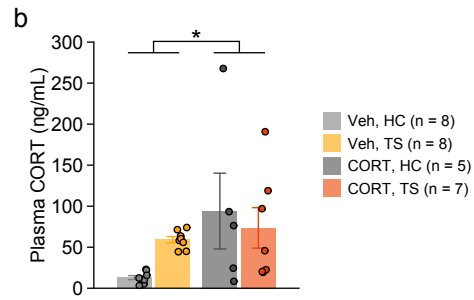

Supplement: Supplementary file 4 — Additional file 4: Supplementary Figure 3. Plasma CORT levels in mice chronically treated with corticosterone. Approximately half of the CORT- and Veh-treated mice were subjected to the tail suspension test. The remaining half of the mice were left undisturbed in their home cages. a Percentage of immobility time in the TS test. b CORT levels in plasma taken from CORT- and Veh-treated mice that were either subjected to the TS or left undisturbed in HC. Values are means ± SEM. *p < 0.05. *p < 0.01 [file 13041_2024_1146_MOESM4_ESM.pdf]

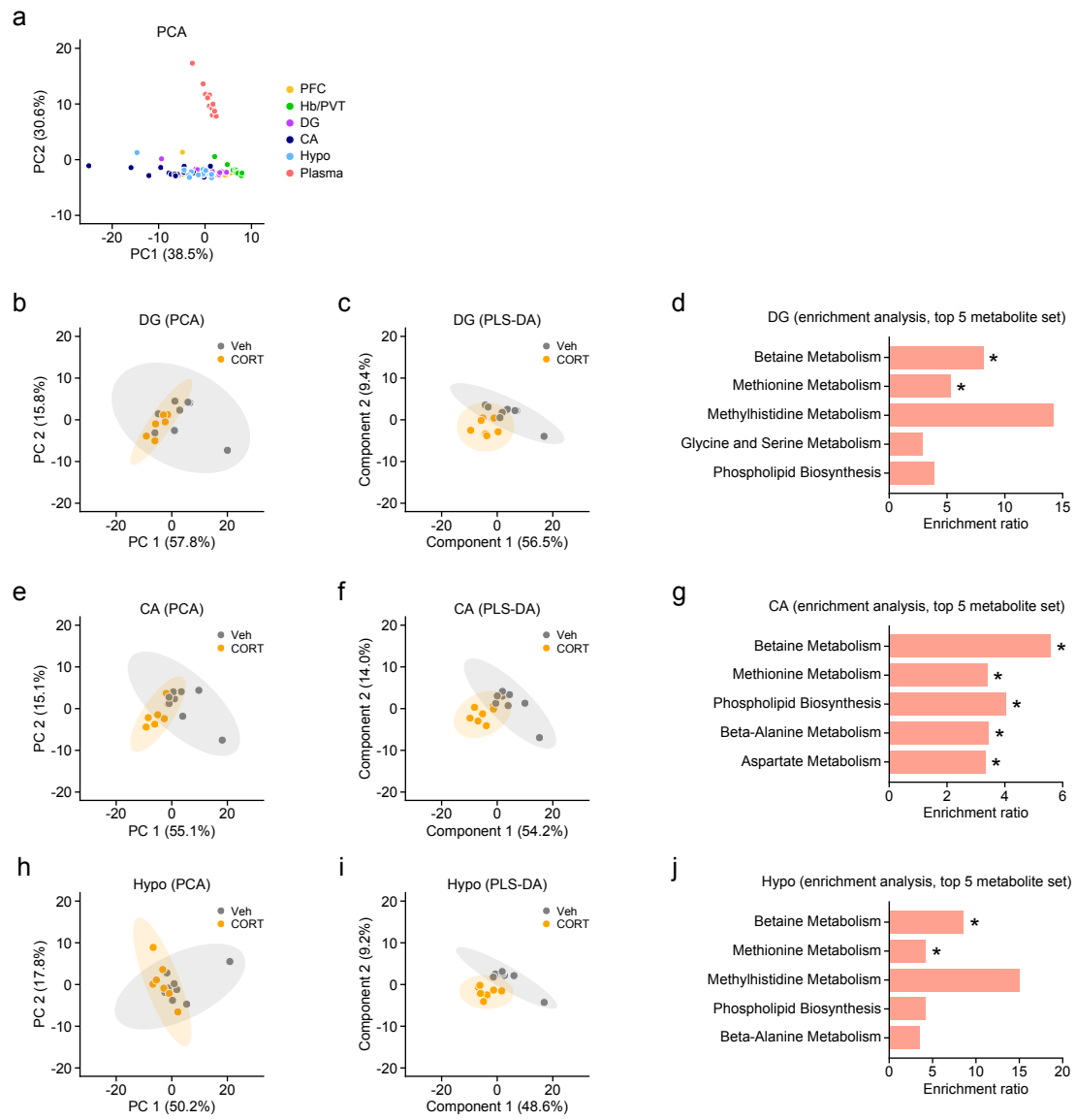

Supplement: Supplementary file 5 — Additional file 5: Supplementary Figure 4. Brain metabolites in mice chronically treated with corticosterone. a Principal component analysis score plot of metabolomic data from the plasma and brain. b, e, h PCA score plots of metabolomic data from CORT- and Veh-treated mice. c, f, i Partial least squares discriminant analysis score plot of metabolomic data. d, g, j Enrichment analysis of metabolites with a VIP value of > 1 in PLS-DA and p-value of < 0.05 in the t-test for comparisons between CORT- and Veh-treated mice. b–d Dentate gyrus. e–g CA in the hippocampus. h–j Hypothalamus. *p < 0.05 [file 13041_2024_1146_MOESM5_ESM.pdf]
